# Reprogramming of the Developmental Program of *Rhus javanica* During Initial Stage of Gall Induction by *Schlechtendalia chinensis*

**DOI:** 10.3389/fpls.2020.00471

**Published:** 2020-05-15

**Authors:** Tomoko Hirano, Seisuke Kimura, Tomoaki Sakamoto, Ayaka Okamoto, Takumi Nakayama, Takakazu Matsuura, Yoko Ikeda, Seiji Takeda, Yoshihito Suzuki, Issei Ohshima, Masa H. Sato

**Affiliations:** ^1^Laboratory of Cellular Dynamics, Graduate School of Life and Environmental Sciences, Kyoto Prefectural University, Kyoto, Japan; ^2^Faculty of Life Sciences, Kyoto Sangyo University, Kyoto, Japan; ^3^Institute of Plant Science and Resources, Okayama University, Okayama, Japan; ^4^Laboratory of Cell and Genome Biology, Graduate School of Life and Environmental Sciences, Kyoto Prefectural University, Kyoto, Japan; ^5^Department of Food and Life Sciences, College of Agriculture, Ibaraki University, Ibaraki, Japan; ^6^Laboratory of Applied Entomology, Graduate School of Life and Environmental Sciences, Kyoto Prefectural University, Kyoto, Japan

**Keywords:** *Rhus javanica*, floral organ development, gall formation, *Schlechtendalia chinensis*, RNA-seq analysis

## Abstract

Insect galls are unique organs that provide shelter and nutrients to the gall-inducing insects. Although insect galls are fascinating structures for their unique shapes and functions, the process by which gall-inducing insects induce such complex structures is not well understood. Here, we performed RNA-sequencing-based comparative transcriptomic analysis of the early developmental stage of horned gall to elucidate the early gall-inducing process carried out by the aphid, *Schlechtendalia chinensis*, in the Chinese sumac, *Rhus javanica*. There was no clear similarity in the global gene expression profiles between the gall tissue and other tissues, and the expression profiles of various biological categories such as phytohormone metabolism and signaling, stress-response pathways, secondary metabolic pathways, photosynthetic reaction, and floral organ development were dramatically altered. Particularly, master transcription factors that regulate meristem, flower, and fruit development, and biotic and abiotic stress-responsive genes were highly upregulated, whereas the expression of genes related to photosynthesis strongly decreased in the early stage of the gall development. In addition, we found that the expression of class-1 *KNOX* genes, whose ectopic overexpression is known to lead to the formation of *de novo* meristematic structures in leaf, was increased in the early development stage of gall tissue. These results strengthen the hypothesis that gall-inducing insects convert source tissues into fruit-like sink tissues by regulating the gene expression of host plants and demonstrate that such manipulation begins from the initial process of gall induction.

## Introduction

Galls are plant tissues or organs formed by hyperplasia (increased cell number) and/or hypertrophy (increased cell size) induced by parasitic or pathogenic organisms including viruses, fungi, bacteria, nematodes, mites, and insects (Mani, 1964). Among galls formed by various organisms, insect galls are extraordinarily complex and highly organized structures comprised of several specialized tissue types (Stone and Schönrogge, 2003; Giron et al., 2016). Insect galls range in complexity from relatively simple mine-galls (Guiguet et al., 2018), open- or folded-type galls such as pit galls, blister galls, and roll galls to complex structures in which the gall-inducing insects are entirely enclosed by plant tissues to form covering galls or mark galls in leaf, stem, and bud (Dreger-Jauffret and Shorthouse, 1992; Guiguet et al., 2019).

The most complex gall structures are generated by gall wasps, gall midges, and gall-inducing aphids in which the galls have extra-floral nectarines, and a coating of hair, spines, and sticky resins (Price et al., 1987; Stone and Schönrogge, 2003; Wool, 2004). The complex insect galls consist of various tissues such as nutritive and protective tissues. The nutritive tissues consist of callus cells and vascular cells, which transport nutrients to the callus; the tissues are ingested by gall-inducing insects. The protective tissues (sclerenchyma) are composed of lignified cells arranged as a layer on the outside of the nutritive tissues and function as a physical shelter against natural enemies and outside environment.

Several lines of evidence indicate that many gall-inducing insects have the potential to precisely secrete effectors into plant tissues using their mouthparts or ovipositors, and such effectors are likely to play a central role in gall induction (Sopow et al., 2003; Matsukura et al., 2009; Stuart et al., 2012; Giron et al., 2016). Thus, gall-inducing insects are believed to manipulate plant developmental programs to generate complex gall structures by secretion of certain chemical compounds in plants (Miles, 1968), and this idea has been supported by histological observations and physiological analyses of insect galls (for a review Giron et al., 2016). The most important characteristic of insect galls is their function as a sink for insect nutrition (Rohfritsch and Shorthouse, 1982). The existence of insect galls near the source organs redirects the flow of plant resources such as carbohydrates, lipids, proteins from the original sink organs to the induced galls. Thus, gall formation results in development of a stronger sink of nutrients for gall-inducing insects than the original sink organs such as buds, flowers, fruits, and storage roots (Weis and Kapelinski, 1984; McCrea et al., 1985; Burstein et al., 1994; Larson and Whitham, 1997).

Darwin (1868) pointed out that the shapes of some complex insect galls resemble flowers or fruits. Indeed, many remarkable flower- and fruit-like traits are observed in insect galls, in particular those that are induced by gall midges and cynipids on various plant species (Rohfritsch and Shorthouse, 1982), suggesting that the formation of gall tissues is similar to the development of flowers or fruits (Kurosu and Aoki, 1990; Ferreira and Isaias, 2014). Recently, Schultz et al. (2018) reported that the gene expression pattern during Phylloxera leaf-gall development is similar to that during carpel development. These results indicate that the parasite may, at least partly, hijack the processes of flower development during gall formation (Schultz et al., 2018), supporting the hypothesis that flower- or fruit-like galls are generated by manipulation of flower development, although the initial process of induction of gall tissues in vegetative tissues is still largely unknown.

Aphids are small phloem sap-feeding insects belonging to the super family Aphidoidea, which embraces approximately 5,000 species in nature (Blackman and Eastop, 2007). Of these, no more than 10% of the aphid species can induce apparent galls on their host plants (Wool, 2004). Like other aphids, the gall-inducing aphids have complicated life cycles, in which a fundatrix or stem mother emerges from a fertilized egg in spring, and initiates the induction of a gall on the primary host plant. Then, the fundatrix parthenogenetically produces offspring inside the gall, and this parthenogenetic production is continued over several generations in particular aphid taxa. In summer or early autumn, winged adults appear and exit from the gall for migrating to the secondary host plant, where they spend several generations in autumn and winter (Wool, 2004; Aoki and Kurosu, 2010). A gall-inducing aphid, *Schlechtendalia chinensis*, induces large, single-chamber galls called horned galls on the leaf wings of several *Rhus* species (Anacardiaceae) in China, Korea, Taiwan, Malaysia, and Japan (Blackman and Eastop, 2007). Galls are first induced when the fundatrix of *S. chinensis* feeds on the adaxial side of the leaf wings. After the fundatrix is enclosed in the gall, the gall is enlarged quickly to form large horned galls with forked structures. During gall development, drastic morphological rearrangement occurs in the leaf wing tissues, in which the palisade tissues of the galled leaf wings are reorganized and replaced by parenchyma cells, and galled zones connect to non-galled zones by newly formed vascular bundles (Liu et al., 2014). Such complexity both in the developmental process and in the structure of *S. chinensis* galls implies that modified but well-organized host-plant gene networks could be incorporated in the process of gall development. However, the underlying molecular mechanisms contributing to the gall formation are largely unknown.

In this study, we performed RNA-sequencing-based comparative transcriptomics of a host plant, *R. javanica*, to understand the molecular characteristics of the early phase of gall development induced by *S. chinensis*. We found that there was no clear similarity in the global gene expression profiles between the gall tissue and other tissues. The genes involved in the phytohormone metabolic and signaling pathways, abiotic and biotic stress responses, and organ development were significantly upregulated, whereas photosynthetic genes were dramatically downregulated. These results imply that the gall-inducing aphid manipulates the plant reproductive programs to convert source tissues into fruit-like sink tissues during the initial process of gall induction.

## Materials and Methods

### Plant Materials

**FIGURE 1:**
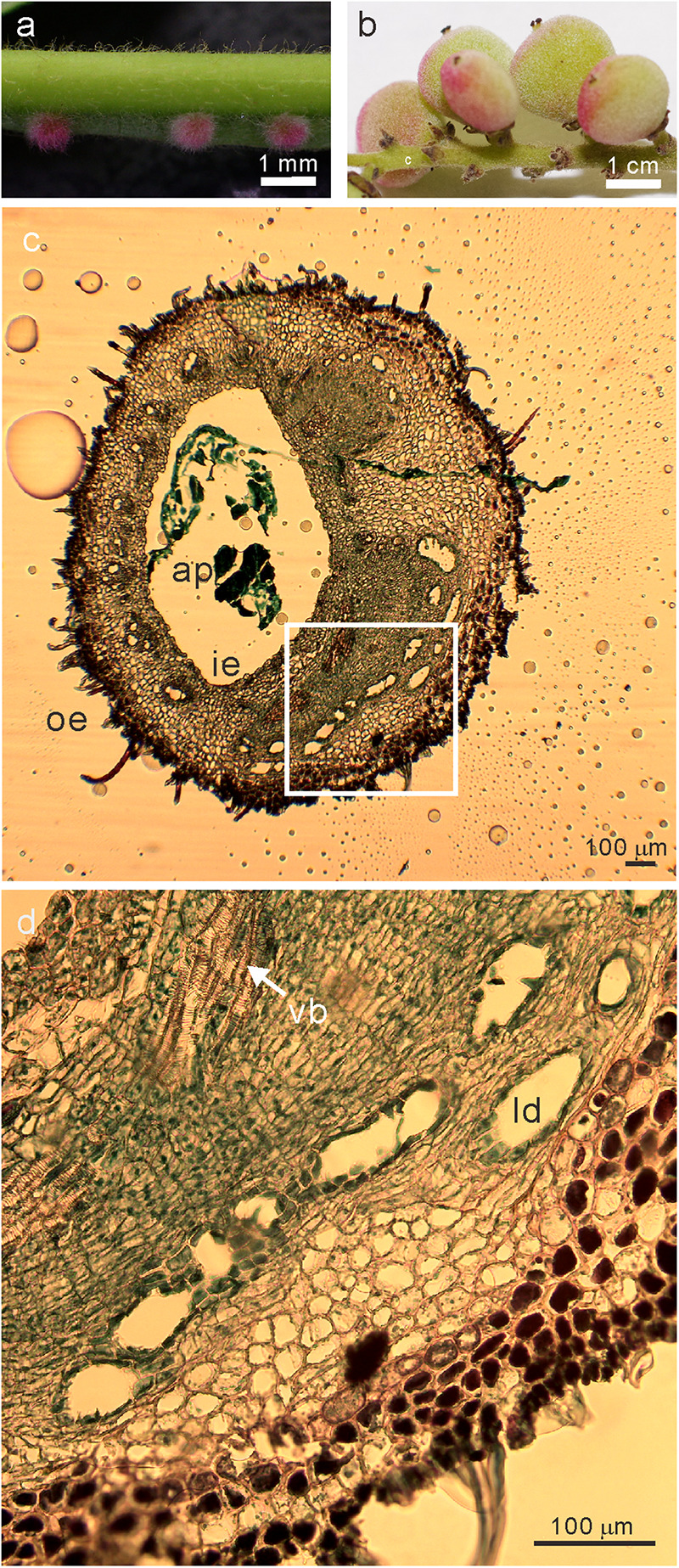
Images of the sampled **(a)** galls at the early developmental stage of (phase 4) and **(b)** fruits **(c)** transverse section of stage 4 galls **(d)** the magnified image (white square in **c**) of *R. javanica*. Abbreviations: ap; aphid; ie, inner epidermis; oe, outer epidermis; ld, latex duct; vb, vascular bundles.

The phase 4 of developmental stage of galls (Liu et al., 2014) (collected in May 22, 2017, Figure 1a), young leaves (collected in May 22, 2017), flowers (collected in September 9, 2017), and fruits (collected in September 28, 2017) (Figure 1b) of *R. javanica* were collected from a natural plantation located in the Kyoto Prefecture of Japan (35°06′00.83″N 135°72′86.94″E).

### RNA Extraction, Library Construction, and RNA-Seq Analysis

Total RNA was extracted from the young leaves, female flowers, fruits, and gall tissues with an RNeasy Plant Mini Kit (QIAGEN). RNA-seq libraries were prepared using the Illumina TruSeq Stranded RNA LT Kit (Illumina, CA, United States) according to the manufacturer’s instructions. Three independent RNA samples for each tissue were used for the analysis. The qualities of the prepared libraries were checked using the QuantiFluor dsDNA System and Agilent High Sensitivity DNA Assay (Agilent, CA, United States). The pooled libraries were sequenced on the NextSeq500 sequencing platform (Illumina, CA, United States) and paired-end reads were obtained. Then, the obtained reads were assembled into transcriptome contigs using Trinity with the default settings. Blastx searches of the obtained contigs against non-redundant protein sequences from GenPept, SwissProt, PIR, PDF, PDB, and NCBI RefSeq (nr) databases using the DIAMOND software (Buchfink et al., 2015) were conducted to find similar protein sequences. Each contig was classified into a taxon group based on the top hits of the blastx results and NCBI taxonomy lineage data. Finally, the contigs classified into the Virdiplantae (plant) kingdom were extracted as *R. javanica* reference transcript contigs to exclude contigs from aphids or other contaminants. RNA-seq analysis of *R. javanica* tissues was biologically repeated at least three times per each tissue sample (Supplementary Table S1).

### Gene Expression Profiling With RNA-Seq Data

The obtained reads were mapped to the *R. javanica* reference transcript contigs using the Burrows-Wheeler alignment tool (BWA)^1^. The count data were subjected to the trimmed mean of *M*-values normalization in EdgeR. Multi-dimensional scaling was performed by calculating the log-fold changes between the accessions and by using differentially expressed genes (DEGs) to compute distances in EdgeR with the plotMDS function. Transcript expression profiles and DEGs were defined with the EdgeR general linear models approach (Robinson et al., 2010). The threshold for DEGs was a log-fold change of >2 and a false discovery rate of <0.01.

### Cloning of cDNAs From *R. javanica* Tissues and Quantitative Reverse Transcription PCR Analysis

The gall, young leaf, flower, and fruit samples were frozen in liquid nitrogen. The total RNA was isolated using the NucleoSpin RNA Plant and Fungi Kit (Takara), and the cDNA library construction was performed using the ReverTra Ace qPCR RT Master Mix (TOYOBO) as per the manufacturer’s instructions. The same amount of cDNA was used as a template for the qPCR, which was performed with the THUNDERBIRD SYBR qPCR Mix (TOYOBO) and gene-specific primers. UBQ10 was used as an internal control for normalization. The primers used in this study are listed in Supplementary Table S2.

### Quantitative Analysis of Indole-3-Acetic Acid and Cytokinins

The endogenous levels of the indole-3-acetic acid (IAA) and cytokinins (CKs) in the whole *S. chinensis* bodies were quantitatively analyzed according to Tanaka et al. (2013). Briefly, the endogenous levels of IAA and cytokinins in the aphids were analyzed by extracting the samples that were spiked with stable isotope-labeled internal standards ([^2^H_5_]tZ, [^2^H_5_]tZR, [^2^H_6_]iP, [^2^H_6_]iPR, and [^13^C_6_]IAA), pre-purifying them with solid-phase extractions, and quantifying them by liquid chromatography/tandem mass spectrometry (3200 QTrap, AB Sciex).

tZ, iP, IAA contents in *R. javanica* leaves and galls were determined according to Yamane et al. (2019) with minor modifications. Briefly, leaf and gall samples (approximately 100 -200 mg per sample) were collected and frozen in liquid nitrogen, and the weight of each tissue was measured. Then the samples were ground and subjected to extraction in 80% acetonitrile and 1% acetic acid containing stable isotope-labeled compounds for internal standards [D_5_-tZ, D_6_-iP, _13_C_6_-IAA] (OlChemim, Czech Republic). After sample purification by HLB and MCX columns (Waters), Phytohormones were analyzed with a 6410 Triple Quad LC/MS System (Agilent Technologies Inc., United States) equipped with a ZORBAX Eclipse XDB-C18 column and an XDB-C8 Guard column (Agilent Technologies Inc.), and peak areas were determined using MassHunter Workstation software (vB.04.00; Agilent Technologies). Four independent samples were analyzed for calculation of averages and standard deviations.

### Histological Analysis and RNA *in situ* Hybridization

Young gall tissues were fixed in the fixative solution consisting of 4% (w/v) paraformaldehyde in 1X PBS under vacuum condition until the samples were drawn to the bottom of the tube. After fixation, the sample were dehydrated through a graded ethanol series, and then followed by a D-Limonene series, and embedded in Paraplast Plus (Sherwood Medical). Microtome sections (4 μm) were deparaffinized in D-Limonene, and rehydrated through a graded ethanol series. In the case of histological observation, the sections were stained with Hematoxylin-Safranin-Fast Green FCF.

Full-length cDNA of RjKNAT6 (KNAT6 of *Rhus javanica*) was cloned into the pENTR vector, and then was subcloned into pGEM11 vector by the Gateway system. Labeled RNA probes were synthesized using *in vitro* transcription in the presence of Digoxigenin-11-UTP by RNA polymerases T7 or SP6 (DIG RNA labeling Mix, Roche). Samples were washed twice in PBST (1X PBS plus 0.1% (v/v) Tween-20) for 10 min and then incubated with 1 μg ml^–1^ proteinase K (Roche) for 15 min. Digestion was stopped by incubating the samples in 1X PBS plus 0.2% glycine for 5 min and then washing them twice in PBST for 10 min. Samples were washed twice in PBST for 10 min and once in the hybridization solution 50% (v/v) formamide in 2X SSC (20X SSC: 3 M NaCl, 300 mM sodium citrate, pH 7.0 with 1 M HCl) for 10 min, and then preincubated in the same solution for 1 h at 50°C. The hybridization step was performed overnight at 42°C by incubating samples in supplemented hybridization solution containing a cocktail of denatured (80°C for 2 min) labeled RNA probes (20–100 ng per ml of the hybridization solution). Samples were washed: three times (10, 60, and 20 min) in a solution of 50% (v/v) formamide. Thereafter samples were incubated with a mixture of the selected primary antibodies (Chicken anti-digoxigenin, Immunology Consultant Laboratory) diluted (1:100) in (PBST + BSA), for 2 h at RT under gentle shaking. Subsequently, samples were washed three times for 10 min in PBST, once for 30 min in PBST plus BSA and then incubated with a mixture of the secondary antibodies (Alexa Fluor dyes 555 Goat Anti-Chicken, INVITROGEN) diluted (1:100) in PBST plus BSA overnight at RT in the dark. After incubation samples were washed twice for 15 min in PBST under gentle shaking in the dark. Fluorescence and differential interference contrast (DIC) images were obtained using Leica TCS SP8 laser scanning confocal microscope. The captured images were processed using Leica LAS X.

## Results

### Transcription Factors Involved in Meristem Formation and Flower Development Were Expressed in Gall Tissues

Liu et al. (2014) reported the histological analysis of the developmental process of *R. chinensis* gall; they categorized the developmental process of the gall into six different phases. To investigate the changes in the gene expression profile during the early phase of gall development, we isolated total RNA from various tissues including entire galls in the phase 4 of development (about 1 mm diameter) in which the gall is completely closed (Figure 1a and Supplementary Figure S1a), young leaves (Supplementary Figure S1b), female flowers (Supplementary Figure S1c), and fruits (Figure 1b). The cross-section of the phase 4 gall revealed that the inner and outer epidermis had two to three layers and the parenchyma cells were well developed between the outer and inner epidermal cell layers with vascular bundles and latex ducts (Figures 1c,d). Phase 4 galls enlarged slowly and contained 1–2 aphids inside of a gall, then in phase 5, the size of the gall increased quickly from August to late September, and finally the horn-like or fork shaped galls were formed (Liu et al., 2014). According to the enlarging gall size, the number of aphids inside of galls increases exponentially (Supplementary Figure S2).

Paired-end reads for the gall, young leaf, flower, and fruit tissues were obtained by RNA-seq (Supplementary Table S1). *De novo* assembly of all the reads yielded 265,145 transcript contigs by Trinity with N50 and average lengths of 1842 and 905.3 nt, respectively. The reference transcript contigs for *R. javanica* were extracted from the raw assembled contigs based on the blastx results against known protein databases, and their N50 and average lengths were 2267 nucleotides and 1331 nucleotides, respectively. Based on the N50 length, which is an indicator of assembly quality, we confirmed that the quality of the *de novo* assembly was sufficient for the subsequent analyzes (Supplementary Table S2).

We aligned all single reads to the contigs and compared the number of DEGs in the galls, flowers, and fruits to those in the young leaves. First, we performed a principal component analysis to compare the gene expression profiles of the gall, leaf, flower, and fruit tissues of *R. javanica*. The eigen values of the two components were greater than 1, and the first component and second component explained 41.4 and 34.6% of the variation, respectively (Supplementary Figure S3a). The factorial map of the principal component analysis showed that the four dots corresponding to each tissue were widely distributed in the graph (Supplementary Figure S2b). These results suggested that there was no clear similarity in the global gene expression profiles between the gall tissue and other tissues.

**FIGURE 2:**
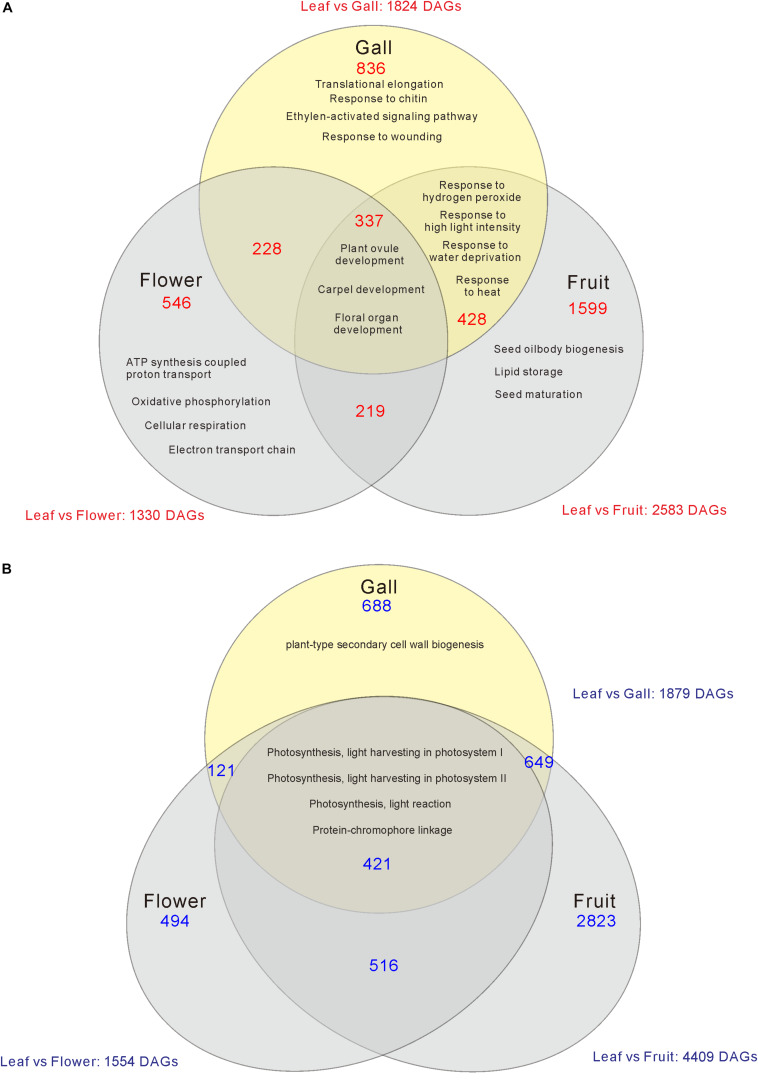
Venn diagram analysis of the number of increased **(A)** or decreased **(B)** differentially expressed genes (DEGs) in the gall, flower, and fruit tissue compared with the young leaves. The numbers in each region indicate the DEGs in each tissue. Overlapping regions of the Venn diagram indicates shared DEGs among corresponding groups. Descriptions in each region indicate typically enriched gene ontology categories of the corresponding groups.

**FIGURE 3:**
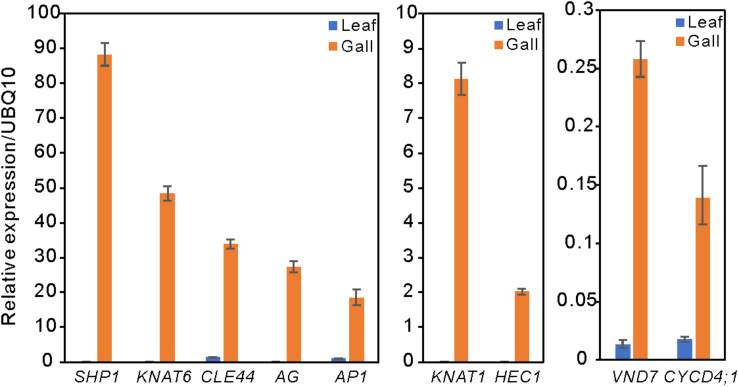
Relative expression levels of several upregulated DEGs in the leaf and gall tissues. The gene expression levels were analyzed by quantitative reverse transcription PCR. UBQ10 served as the internal expression control. Experiments were repeated three times using three biologically distinct samples, and gave similar results. The data is represented as the mean ± standard deviation (Four technical replicates).

Compared with the transcripts for young leaves, the transcripts for the gall, flower, and fruit tissues showed upregulation of 1829, 1330, and 2583 DEGs, and downregulation of 1879, 1554, and 4409 DEGs, respectively (log-fold change of >2 and a false discovery rate of <0.01) (Figure 2 and Supplementary Tables S3, S4). As the *R. javanica* genome has not yet been read and no functional annotation exists, we assigned *R. javanica* transcripts to *Arabidopsis thaliana* orthologs using functional annotations from The *Arabidopsis* Information Resource (TIAR). To assess the similarity between the *R. javanica* and *Arabidopsis* orthologs, we cloned and sequenced several *R. javanica* full-length transcripts (*AG, AP1, CLE41/44), SEP2, CYCD4;1, and SEOR1)* according to the deduced sequences assembled by Trinity, and then compared them with the full-length sequences of the *Arabidopsis* counterparts. The cloned transcripts have considerable similarity with the *Arabidopsis* counterparts (Supplementary Figure S4), implying that most of the *R. javanica* transcripts could be correctly assigned to the *Arabidopsis* orthologs. To evaluate the DEGs identified by RNA-seq, we measured the differences in the expression levels of several of the upregulated genes by quantitative real-time PCR and found that *SHP1, KNAT6, CLE44, AG, AP1, KNAT1, HEC1, VND7*, and *CYCD4;1* were considerably upregulated in the gall tissue (Figure 3).

### Genes Involved in Meristem Formation and Floral Organ Development Were Upregulated in the Gall, Flower, and Fruit Tissues

**FIGURE 4:**
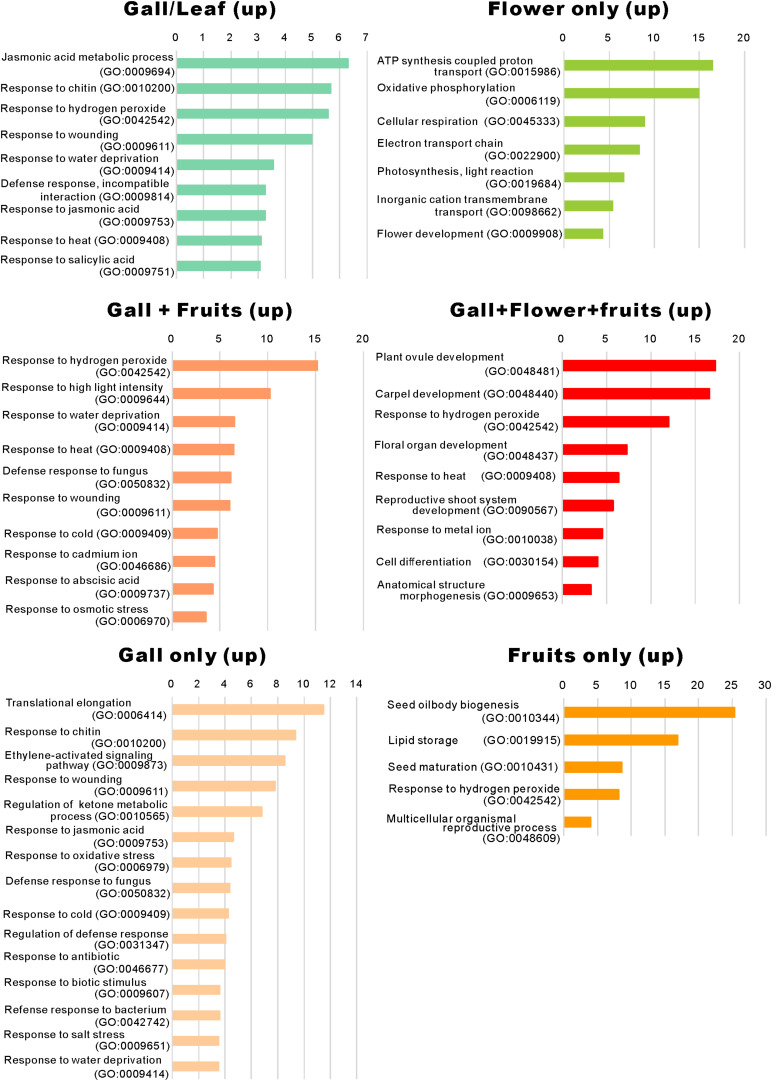
Gene ontology term enrichment of the upregulated genes. The upregulated differentially expressed genes in Figure 2A were subjected to the enrichment analysis (Mi et al., 2019) using the TAIR website (https://www.arabidopsis.org/tools/go_term_enrichment.jsp).

**FIGURE 5:**
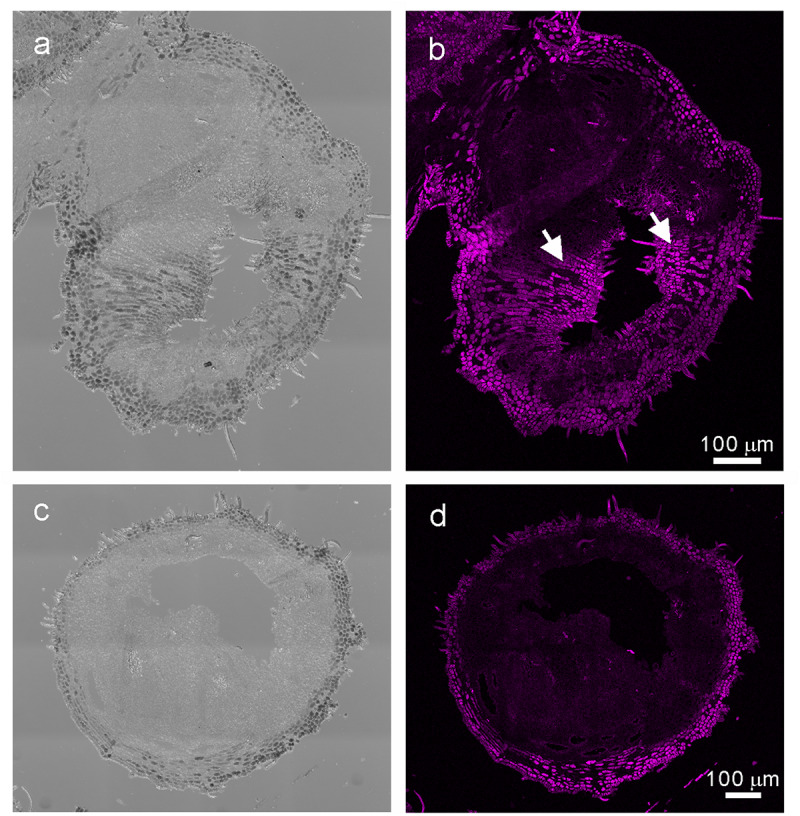
*In situ* hybridization analysis of *R. javanica* KNAT6 in the early developing stage of gall. **(a)** Differential interference contrast (DIC) image of cross-section of the developing gall hybridized with RjKNAT6 anti-sence probe. **(b)** Fluorescent image of the developing gall hybridized with RjKNAT6 anti-sence probe. **(c)** Differential interference contrast (DIC) image of cross-section of the developing gall hybridized with RjKNAT6 sence probe. Arrows indicate the positive signals of the anti-sense probe. **(d)** Fluorescent image of the developing gall hybridized with RjKNAT6 sense probe. The images were taken at least three biologically distinct samples and gave the same results. Scale bars: 200 μm.

When comparing the genes upregulated in the galls with those upregulated in the flowers and fruits, we found that expression of 337 genes was increased in the gall, and in the floral and reproductive organs. Gene ontology (GO) term enrichment analysis of these upregulated genes revealed that the genes assigned to the GO categories of the floral organ development (GO: 0048481, 0048440, 0048437, and 0090567) were enriched by over 5-fold (Figure 4). Of these, several genes encoding transcription factors involved in the regulation of floral organ morphogenesis were upregulated in the early development stage of gall tissue. For example, the upregulated genes included a floral integrator (*LFY*), class-1 *KNOX* genes (*KNAT1/2/6, and STM*), and MADS-box-type transcription factors (*SEP1, SEP2, SEP3, AP1, AP3 AG, TT16*, *FUL*, and *SHP1*) (Supplementary Table S5). *In situ* hybridization analysis revealed that *KNAT6* was predominantly expressed in the parenchyma cells of developing gall tissues (Figure 5).

### Phytohormone Metabolic and Signaling Pathways Were Activated in the Gall Tissue

In the *R. javanica* gall tissue, genes involved in the auxin- (*IAA17, PILS1, GH3.1, GH3.3, WRKY23*, and *PBP1*), ethylene- (*ERF017, ERF022, ERF13, ERF72*, and *ERF109*), and abscisic acid- (*NHL6*, *MAPK3, AHG1, CBF4, ABR1*, and *RDUF2*) response pathways were significantly upregulated (Supplementary Tables S5, S7), whereas genes belonging to the GO category “response to cytokinin (GO:0009735)” were downregulated (Supplementary Table S6), suggesting that several phytohormone signaling pathways may be activated by the actions of the gall-inducing aphid. Since active phytohormones such as indole-3-acetic acid (IAA), abscisic acid, and cytokinins (CKs) have been identified in several gall-inducing insects (Mapes et al., 2001a, b; Dorchin et al., 2009; Yamaguchi et al., 2012; Tanaka et al., 2013), we reasoned that *S. chinensis* would also produce phytohormones for gall induction on *R. javanica* leaves. Therefore, we measured the contents of IAA and CKs in the whole aphid bodies, and identified a considerable amount of IAA (718.9 ± 269.0 ng/g FW) and CKs, particularly iP (5.106 ± 1.503 ng/g FW), iPA (7.726 ± 1.451 ng/g FW), and tZR (7.726 ± 1.451 ng/g FW) in the whole aphid body (Table 1). In contrast, the concentrations of these phytohormones in gall and leaf tissues were lower than those in the aphid body (Supplementary Figure S5).

**TABLE 1:** Endogenous phytohormone contents in *S. chinensis*.

Plant hormone	Average (ng/g)
IAA	718.9 ± 269.0
iP	5.106 ± 1.503
iPA	7.726 ± 1.451
tZ	0.0220 ± 0.0118
tZR	6.832 ± 0.8960

*The results are given as the mean ± standard deviation from five replicates. Abbreviations: IAA, indole-3-acetic acid; iP, isopentenyladenine; iPA, isopentenyladenosine; tZ, trans-zeatin; tZR, trans-zeatin riboside. Experiments were repeated at least three times using three biologically distinct samples.*

### Genes Involved in the Secondary Cell Walls, Vascular Tissue, and Callus Formation Were Highly Upregulated in the Gall Tissue

During the gall growth process, the latex ducts and vascular elements become denser in the inner gall layer, the outer epidermal cell layer hardens due to the construction of a lignified secondary cell walls, and the palisade tissue of the galled leaf wings is reorganized and replaced by parenchyma cells (Liu et al., 2014). In this study, we observed that the latex ducts and vascular bundles emerged, and the outer epidermal cell layer began to harden by lignification during the early developmental phase of the galls (Figures 1c,d). In this stage, the genes involved in cell wall synthesis (e.g., *CSLD2/6*, *CESA8*, *XTH4*, and *CEL2*), production of lignin and suberin deposition (e.g., *CAD5, PRX25, CYP84A1, PRX72*, *MYB58*, *FAR4*, and *CER9*), and vascular tissue morphogenesis (e.g., *CLE44, SEOR, VND7*, and *TDR*) were upregulated (Supplementary Table S7). The changes in these genes might reflect the morphological changes in the early stage of gall development.

### Abiotic and Biotic Stress-Response Pathways Were Activated in the Gall Tissues

We identified that a significant number of genes related to “the jasmonic acid metabolic process” (GO: 0009694) and “response to jasmonic acid” (GO: 0009753), “response to chitin” (GO: 0010200), “response to hydrogen peroxide” (GO: 0042542), “response to wounding” (GO: 0009611), and “response to salicylic acid” (GO: 0009751) were considerably enriched only in the developing gall tissues (Figure 5). After a detailed analysis of these genes, we found that the expressions of *AOC3/4*, *JAZ1/2/8/10*, *CYP94C1*, and *JOX2* were dramatically upregulated in the developing gall tissues. Also, a significant number of genes involved in plant–pathogen interactions were upregulated in the gall tissues. In particular, transcription factors related to PAMP-triggered immunity (e.g., *WRKY33, WRKY40, MYB51*, and *TGA9*) were highly expressed in the gall tissues (Supplementary Table S7). Additionally, the expression of a significant number of abiotic stress-responsive genes, including those that respond to water deprivation (GO: 0009414) and heat (GO: 0009408), was increased in the gall tissues (Figure 4). Most of these genes (e.g., *RD17, LTI45, ERD14, HSFB2A, DDF1, LSR3*, and *ERD7*) respond to drought and heat stresses.

### The Changes in Expression of Photosynthetic Genes and Transporter Genes Between Leaf and Gall Tissues

**FIGURE 6:**
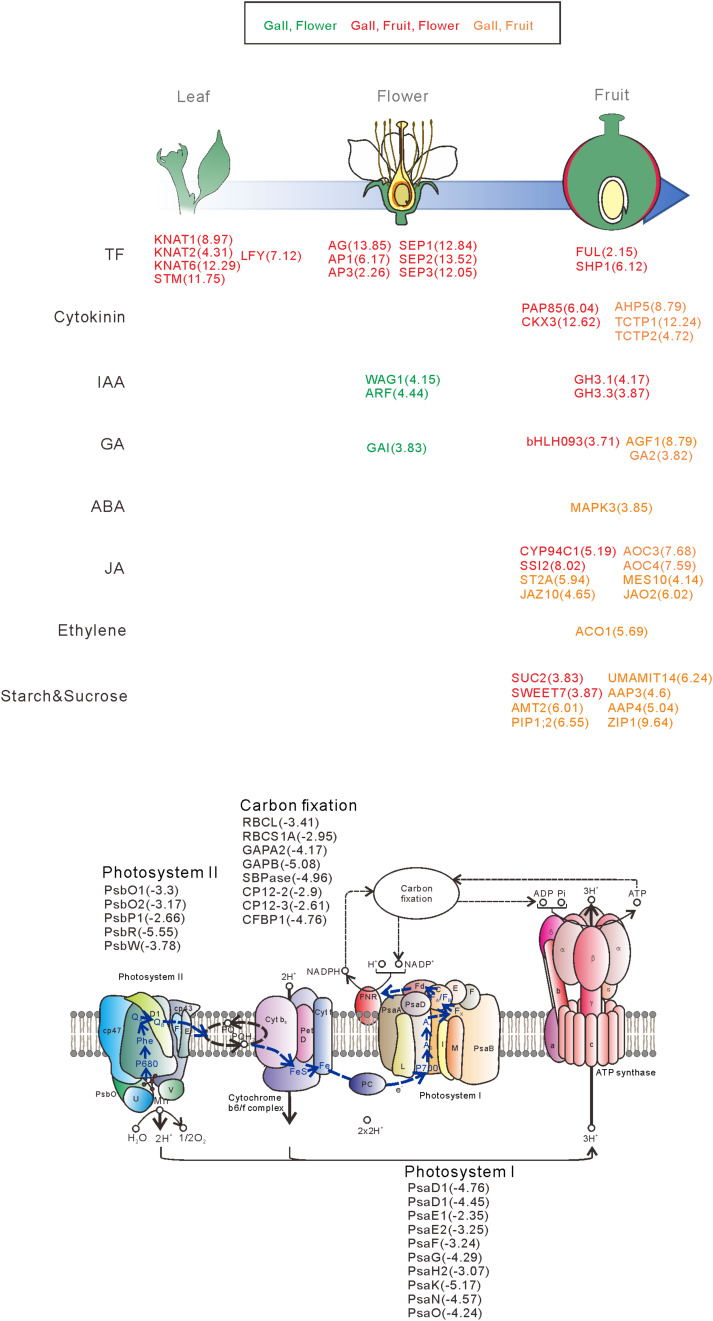
Overview of the representative genes expressed in the galls, flowers, and fruit. The genes are categorized by the developmental stage, the role of the transcription factors, different hormonal signals, and transporters. The numbers in parentheses represent the log-fold change values of the differentially expressed genes compared with the young leaves. Upregulatd genes in gall and flower (Green), gall, fruit, and flower (red), gall and fruit (orange).

We next categorized the 3809 downregulated genes and found that the genes belonged to GO categories related to photosynthesis (GO: 0097868, 0018298, 009769, 0015977, and 0015995) (Supplementary Tables S4, S6). For instance, the components of photosystem I (*PsaD, PsaE, PsaF, PsaG, PsaH, PsaK, PsaN*, and *PsaO*), photosystem II (*PsbO, PsbP, PsbR*, and *PsbW*), and carbon fixation (*GAPA2*, *RBCS1A*, *SBPASE*, and *CFBBP1*) were dramatically downregulated (Figures 2, 6 and Supplementary Table S4). In contrast, the transcripts of various transporters including amino acid transporters (*UMAMIT14*, and *AAP3/4*), sugar transporters (*SUC2*, *SWEET7*), metal transporters (*AMT2*, *ZIP1*), and water transporters (*TIP1;3*, *PIP1;2*) were increased in the gall tissues (Figure 6 and Supplementary Table S7).

## Discussion

### Transformation From Source to Sink Tissues During Gall Formation in the Leaf Wings

One of important characteristics of galls is their function as sinks for insect nutrients (Rohfritsch and Shorthouse, 1982). The existence of insect galls near the source organs changes the flow of plant resources by partially blocking and redirecting the resources from the original sink organs to the galls. Thus, gall formation results in making a stronger sink than plant sink organs such as buds, flowers, and fruit (Weis and Kapelinski, 1984; McCrea et al., 1985; Burstein et al., 1994; Larson and Whitham, 1997). In *R. javanica*, galls start to develop on the leaf wing when the fundatrix of *S. chinensis* feeds on the surface of the leaf wing. In the early stage of gall development, the feeding site on the leaf wing tissue grows abnormally to form hyperplastic tissues, in which the outer epidermal layer of the gall is covered with denser trichomes and is lignified to form a rigid structure. The palisade tissues of the leaf wing are reorganized and replaced by dedifferentiated parenchyma cells, and the latex ducts and vascular elements become denser in the inner gall layer and close to the gall cavity (Liu et al., 2014). Throughout this process, a galling aphid such as *S. chinensis* creates horned galls as a novel source organ on the leaf wing.

In the case of the grape gall formation by phylloxera, the expression of genes associated with “light harvesting and photosynthetic carbon assimilation” strongly decreased, whereas that of the transcripts associated with “sucrose mobilization” and “glycolysis and fermentation” considerably increased in gall tissues compared to that in ungalled leaf tissues (Nabity et al., 2013). In this study, we revealed that the photosynthesis-related genes involved in the photosystem I (GO: 0009768), photosystem II (GO: 0009769), and carbon fixation pathway (GO: 0015977) were dramatically downregulated during the early gall development in *R. chinensis*. In contrast, the expression of the genes involved in the translational elongation process (GO: 0006414) increased, suggesting that genes related to *de novo* protein synthesis necessary for secondary metabolites in gall tissues are activated during gall formation. Through a detailed analysis of the upregulated genes involved in this process, we found that a significant number of molecular chaperons, ribosomal proteins, and various transporter genes such as sugar and amino acid transporters were highly expressed in the gall tissues (Supplementary Tables S5, S7).

Pathogens acquire glucose from their hosts, thereby hijacking host sugar efflux systems (Sutton et al., 1999; Voegele et al., 2001). In particular, SUGARS WILL EVENTUALLY BE EXPORTED TRANSPORTERS (SWEETs) sugar transporters have been reported to be utilized by pathogens for the acquisition of sugars. For instance, the rice homologs SWEET11 and SWEET14 are specifically exploited by bacterial symbionts and fungal and bacterial pathogens, indicating that the sugar efflux function of SWEET transporters is probably targeted by pathogens and symbionts for nutritional gain (Chen et al., 2010). *Plasmodiophora brassicae* is the causal agent of clubroot, a severe disease of Brassica crops. The pathogen lives inside roots, and hijacks nutrient sink in infected roots to trigger active sugar translocation between the sugar producing tissues and the clubbed tissues recruiting the SWEET sucrose transporters within developing galls (Li et al., 2018; Walerowski et al., 2018). In this study, we found that the expression of *Arabidopsis* SWEET7 homolog is increased in the early development stage of gall tissues, indicating that the expression of SWEET sugar transporter gene of *R. javanica* is likely activated by the feeding action of *S. chinensis*.

Collectively, the changes in the expression profile during gall formation imply that the cells of the palisade tissues in the leaf wing were reorganized to be de-differentiated into parenchyma cells, thereby losing their photosynthetic and reconstruction cellular functions, to convert the tissues architecture of the leaf wing from source to sink tissues during the gall development process.

### Expression of Abiotic and Biotic Stress-Related Genes in the Gall Tissues

Plants respond to herbivory with the induction of a combination of defense responses such as salicylic acid, jasmonic acid, and ethylene signaling pathways to produce toxins and defensive proteins that target physiological processes in insects (Agrawal et al., 1999; De Vos et al., 2005). Aphid feeding is perceived by plants as pathogenic and herbivory; hence, on sensing the phytopathogens and mechanical damage caused by stylet probing, the plants elicit a defense response that involves both salicylic and jasmonic acid pathways (Kaloshian and Walling, 2005; Thompson and Goggin, 2006; Gao et al., 2007). It has been reported that the other known minor pathways including ethylene, abscisic acid, gibberellic acid, nitric oxide, and auxin are also activated in response to aphid feeding (Smith and Boyko, 2007).

An investigation on the molecular response of gall formation by the gall-inducing aphid *S. chinensis* on *R. chinensis* by comparing expression profiles of leaves and 100-day grown galls has demonstrated that the genes involved in the biosynthesis of secondary metabolites, plant–aphid interactions, and plant hormone signal transduction were highly expressed in galls (Wang et al., 2017). In this study, we compared the expression profile of the early developmental galls with that of the early developmental leaves, and found that the salicylic acid-, jasmonic acid-, and ethylene-response pathways were considerably activated in the gall tissues. In particular, genes related to the wounding response and PAMP-triggered immunity (Supplementary Table S7) were highly expressed in the gall tissues. These defense responses were likely induced by sensing the feeding stress of gall-inducing aphid for protecting the plant body from the aphid’s invasion of plant signaling pathways driven by jasmonic acid, salicylic acid, ethylene, abscisic acid, and gibberellic acid. However, the growth and proliferation of *S. chinensis* inside the gall seemed to be unaffected, which was probably because the aphids adapted to, and overcame the host’s defense systems, suggesting that the gall-growing leaves or plants to become more tolerant against other pathogenic organisms than those are not induced gall.

### Putative Action of Phytohormones of the Gall-Inducing Aphid

During the growing process of the horned gall, caused by the gall-inducing aphid *S. chinensis*, a combination of cell division (hyperplasia) and growth (hypertrophy) occurs in several layers of tissues, inducing the formation of nutritive and protective gall tissues (Liu et al., 2014). It has long been hypothesized that phytohormones produced by gall-inducing insects play a key role in gall formation (Tooker and Helms, 2014). Indeed, active phytohormones such as IAA and CKs have been identified in several gall-inducing insects at various concentrations (e.g., IAA = 60–9000 ng g fw^–1^, iP = 3–350 ng g fw^–1^, iPR = 8–190 ng g fw^–1^, tZ = 2–1300 ng g fw^–1^, and tZR = 0.4–70 ng g fw^–1^) (Mapes et al., 2001a, b; Dorchin et al., 2009; Yamaguchi et al., 2012; Tanaka et al., 2013), and the application of exogenous phytohormones led to induction of gall-like structures in plants (Bartlett and Connor, 2014). We found that the concentrations of IAA and CKs in *S. chinensis* fell within the ranges of those in the gall-inducing insects. The contents of IAA and CKs were considerably higher than those in all and leaf tissues, suggesting that the aphids that may control gall-inducing process on *R. javanica* leaves by producing phytohormones such as IAA and CKs themselves. Given that IAA and CKs are involved in abnormal cell division, cellular enlargement, and differentiation, our results suggest that the phytohormones secreted from the aphids may be involved in the gall formation reported here, although we could not completely rule out the possibility that the phytophormones in aphid are derived from the host plant tissues.

### Gene Expression Similarities Between Gall Formation and Floral Organ Development

Some of insect galls are believed to resemble flowers or fruits in their morphology (Darwin, 1868), suggesting that the developmental program of the reproductive organs of plants may be hijacked and exploited by gall-inducing insects. Recently, Schultz et al. (2018) reported that reproductive genes involved in floral organ development were significantly enriched in the developing galls of wild grapevine that were induced by *Phylloxera*, suggesting that a galling insect utilizes plant reproductive programs during gall development. In the present study, we also found that a significant number of transcription factors involved in floral organ development were upregulated during *R. javanica* gall development. Of these genes, *LFY* is an important master regulator for the transition from vegetative to reproductive phase during meristem development (Blázquez et al., 1997). Constitutive expression of *LFY* under the 35S promoter causes the conversion of indeterminate lateral meristems into flowers and the conversion of the inflorescence meristem into a flower (Weigel and Nilsson, 1995; Siriwardana and Lamb, 2012). *AP1*, *AP2*, and *AG* determine the floral organ identity, and A-, B-, and C-type floral MADS-box genes (Alvarez-Buylla et al., 2010) in combination with the *SEP1/2/3* MADS-box subfamily being required for specifying the “floral state” (Alvarez-Buylla et al., 2010). The *FUL* and *SHP1* genes are also members of the MADS-box transcription factors, and are involved in valve development and differentiation of both the lignified layer and separation layer of the valve margin in the developing ovaries, respectively (Gu et al., 1998; Liljegren and Bowman, 2000).

In addition to these genes, in the present study, we found that the class-1 *KNOTTED1-like homeobox* (*KNOX*) genes (*KNAT1/2/6*, and *STM*) were highly expressed in the developing gall tissues. The *KNOX* genes are found in all higher plant species and encode homeodomain transcription factors similar to those that regulate development in animals (Scofield and Murray, 2006; Hay and Tsiantis, 2010). The class-1 *KNOX* genes in *Arabidopsis* are expressed in the shoot apical meristem (SAM) but not in the lateral organs (Lincoln et al., 1994; Pautot et al., 2001; Lenhard et al., 2002; Dean et al., 2004). It has been reported that the ectopic overexpression of *STM* or *KNAT1* leads to the formation of ectopic knot-like meristematic structures, which results in formation of lobed leaves on the adaxial surface of the leaf (Chuck et al., 1996; Brand et al., 2002; Lenhard et al., 2002). When gall formation begins, the development of ectopic meristematic structures could be made from leaf wing tissues. Similarities in organ structure between the lobed leaves caused by *STM* or *KNAT1* overexpression and the initiation stage of the gall structure imply that the ectopic overexpression of the class-1 *KNOT* genes induced by gall-inducing insects may initiate de-differentiation of the leaf wing cells to generate a meristematic region followed by the formation of an initial gall structure on the leaf wing.

From these results, we propose the following molecular mechanisms of the early stage of gall formation in *R. javanica*: (i) an ectopic meristematic structure is generated by the overexpression of class-1 *KNOX* genes, (ii) the ectopic meristem is converted to floral-like meristem by the expression of *LFY*, (iii) the floral-like meristem develops to form fruit-like gall structures induced by expression of floral regulatory genes, and (iv) during the transformation from leaf to gall tissues, many photosynthetic genes are downregulated, while transporter and secondary metabolic genes are upregulated to change the tissue functions (Figure 6).

## Data Availability Statement

The datasets generated for this study can be found in the NCBI using the following link: https://www.ncbi.nlm.nih.gov/bioproject/?term=PRJDB8441.

## Author Contributions

TH, MS, and IO conceived and designed the study. TH, ST, and AO collected samples and extracted RNA. TH and TN performed qRT-PCR analysis. YS performed a quantitative analysis of indole-3-acetic acid and cytokinins in *S. chinensis*. TM and YI measured the content of various phytohormones in *R. javanica* tissues. TS and SK constructed library, and performed RNA-sequencing. TS, SK, TH, ST, and MS analyzed the data.

## Conflict of Interest

The authors declare that the research was conducted in the absence of any commercial or financial relationships that could be construed as a potential conflict of interest.

## Footnotes

1 http://bio-bwa.sourceforge.net

## Supplementary Material

FIGURE S1 Images of the sampled **(a)** *R. javanica* leaf attaching several phase 4 stage galls at the wing region, **(b)** young leaf **(c)** female flowers.

FIGURE S2 Sizes and numbers of aphids inside of various stages of galls. **(a)** The number of aphids inside of the developmental galls. **(b–e)** Images of the developmental galls corresponding to the plots in **(a)**.

FIGURE S3 Principal component analysis of the gene expression profile. **(a)** Eigenvalues and the cumulative contribution ratio (8%). Bars and open circles represent the eigenvalues and cumulative contribution ratio, respectively. **(b)** The global expression profile of each transcript as principal components 1 and 2.

FIGURE S4 Amino acid sequence alignment of several orthologous genes of *A. thaliana* and *R. javanica*. AP1, APETALA1 **(a)**; SEP2, SEPALLATA2; CLE41, CLAVATA3/ESR-RELATED 41 **(b)**; CYCD4;1, CYCLIND4; 1 **(c)**.

FIGURE S5 Phytohormone contents in *R. javanica gall and leaf tissues*. The results are given as the mean ± standard deviation from five replicates. Abbreviations: tZ, trans-zeatin iP, isopentenyladenine; IAA, indole-3-acetic acid. Experiments were repeated at least three times using three biologically distinct samples.

TABLE S1 Results of RNA-seq analyzes of various *R. javanica* tissues.

TABLE S2 List of primers used for qRT-PCR.

TABLE S3 Transcriptome sequencing and summary statics of *de novo* assembly.

TABLE S4 List of upregulated genes in gall, flower, fruit of *R. javanica*.

TABLE S5 List of downregulated genes in gall, flower, fruit of *R. javanica*.

TABLE S6 Result of GO enrichment analysis of up-regulated gens in gall of *R. javanica*.

TABLE S7 Result of GO enrichment analysis of down-regulated gens in gall of *R. javanica*.

TABLE S8 List of upregulated genes categorized by the biological processes.
