# Supplementary material for: Reprogramming of the Developmental Program of Rhus javanica During Initial Stage of Gall Induction by Schlechtendalia chinensis
Source: Front Plant Sci. 2020 May 15;11:471. doi: 10.3389/fpls.2020.00471 (PMC7243852; doi:10.3389/fpls.2020.00471)
Supplement: FIGURE S1 — Images of the sampled (a) R. javanica leaf attaching several phase 4 stage galls at the wing region, (b) young leaf (c) female flowers. [file Presentation_1.pdf]

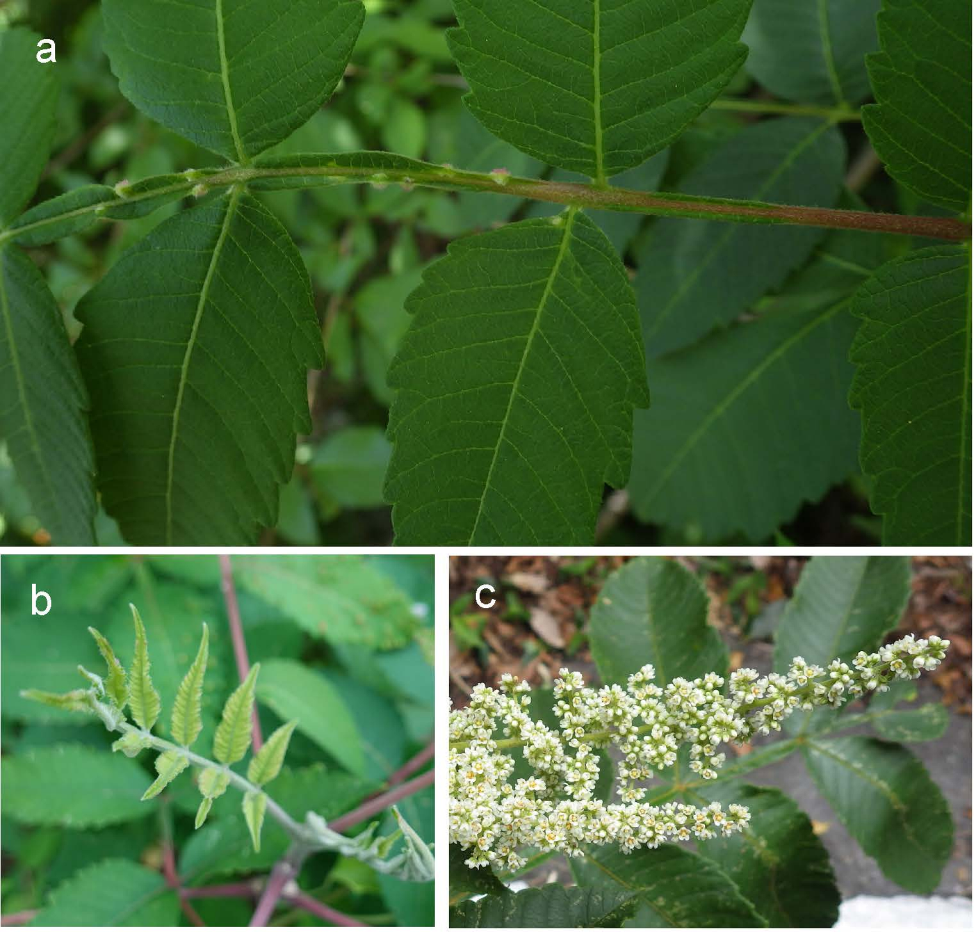

Supplementary Fig. S1

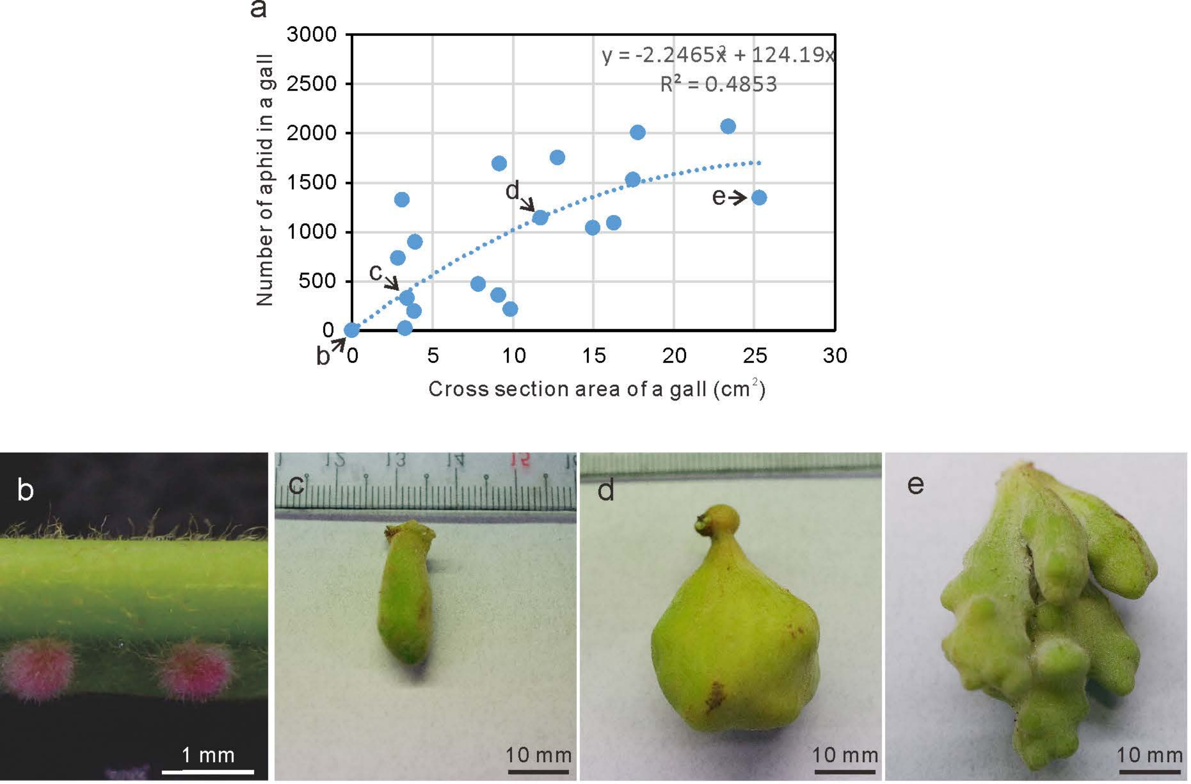

Supplementary Fig. S2

a

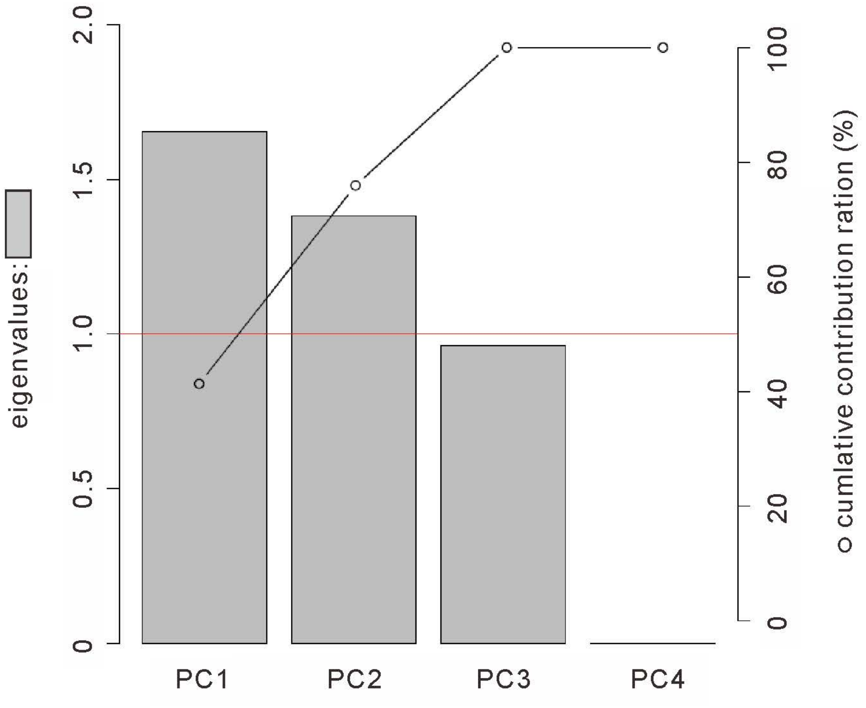

b

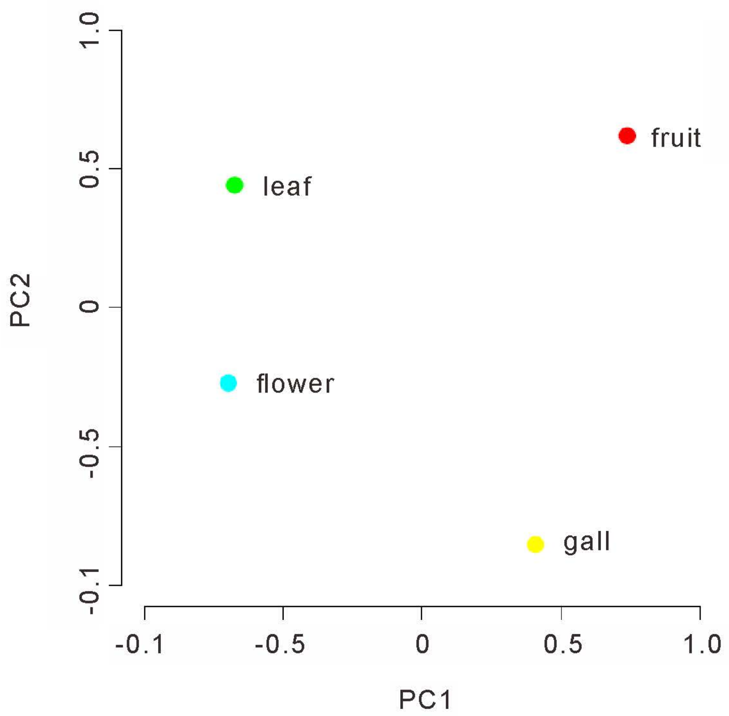

Supplementary Fig. S3

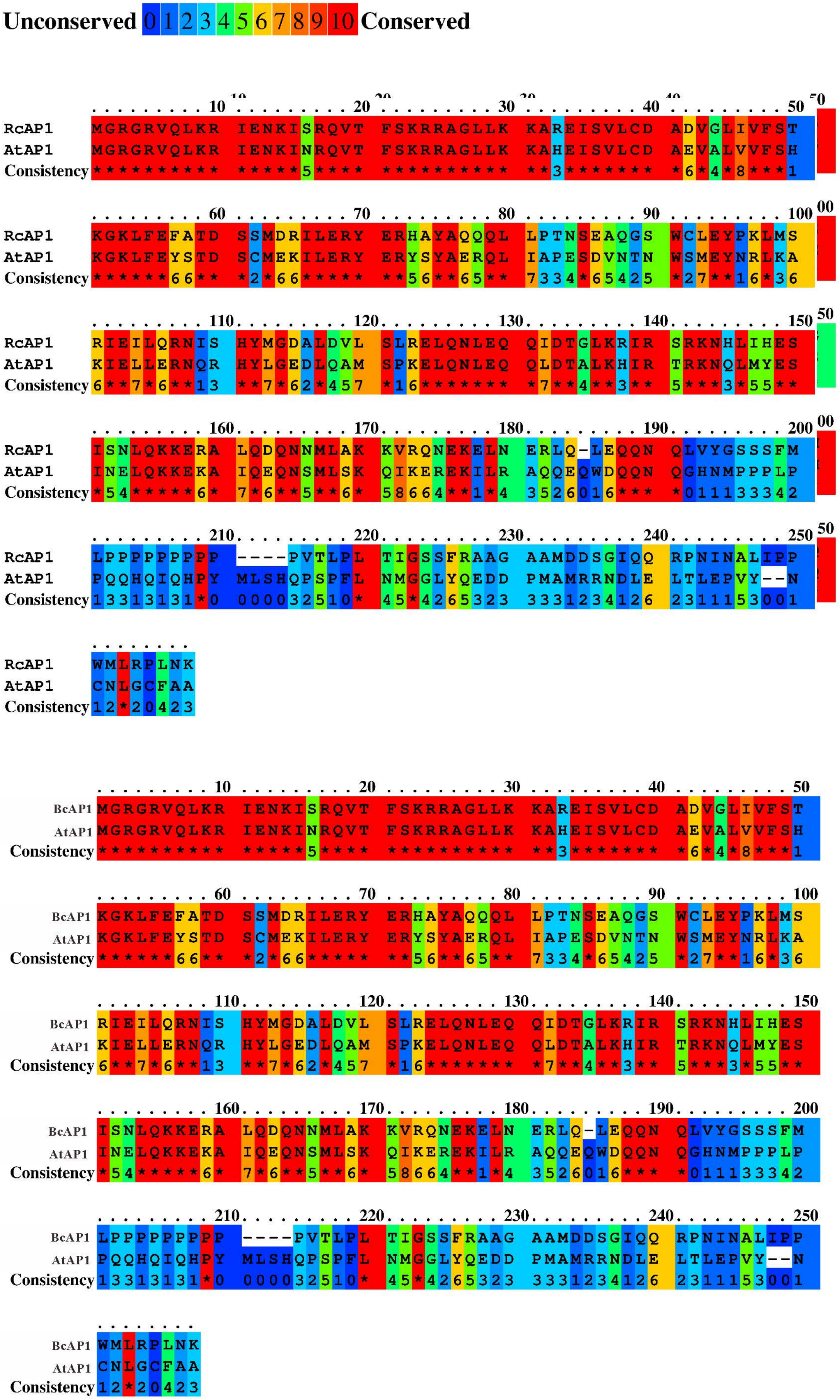

Supplementary Fig. S4a

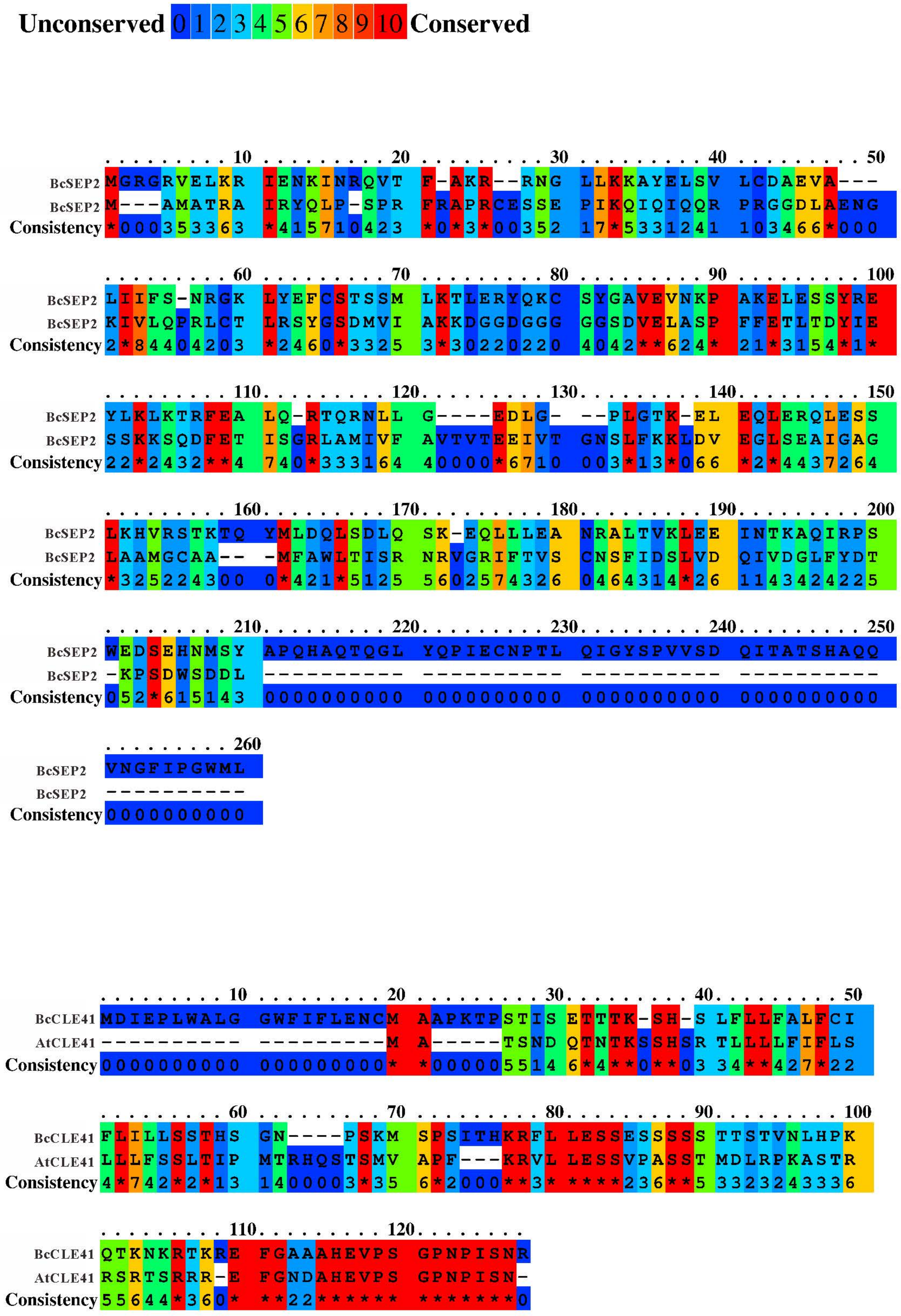

Supplementary Fig. S4b

Unconserved 0 1 2 3 4 5 6 7 8 9 10 Conserved

```
            .........10 .........20 .........30 .........40 .........50
RcCYCD4_1   MAPSFDAALS SLLCTEDNNS IFDDINTDYH GVVVGEFEAT WYHGNHQTRN
AtCYCD4_1   MA--EENLEL SLLCTESNVD D--------- ---------- --EGMIVDET
Consistency **00162312 ******4*14 1000000000 0000000000 003*212344

            .........60 .........70 .........80 .........90 .........100
RcCYCD4_1   QNKRFDDCGD ILPLQSDECL ALMLEKECQH LPNSDYLKRL QIGD-LDLVA
AtCYCD4_1   PIEISIPQMG FSQSESEEII MEMVEKEKQH LPSDDYIKRL RSGDLDLNVG
Consistency 3151212013 42326*6*27 31*6***0** **54**7*** 52**0001*4

            .........110.........120.........130.........140.........150
RcCYCD4_1   RKEAVDWIV- ---------K INAHFDFGPL CAYLSINYLD RFLSAYELPK
AtCYCD4_1   RRDALNWIWK IRGLCRTDRE ACEVHQFGPL CFCLAMNYLD RFLSVHDLPS
Consistency *66*65**00 0000000005 303124**** *21*65**** ****556**4

            .........160.........170.........180.........190.........200
RcCYCD4_1   GKAWMMQLLA VACLSLAAKM EETQVPSCVD LQVGDTKFLF EARTIKRMEL
AtCYCD4_1   GKGWILQLLA VACLSLAAKI EETEVPMLID LQVGDPQFVF EAKSVQRMEL
Consistency **4*57**** *********5 ***6**328* *****35*6* **6585****

            .........210.........220.........230.........240.........250
RcCYCD4_1   LVLSTLRWRM QAITPFSYID YFIRKISDGD QIPLRTLFLS SIQLIISTIK
AtCYCD4_1   LVLNKLKWRL RAITPCSYIR YFLRKMSKCD QEPSNTLISR SLQVIASTTK
Consistency ***53*6**7 5****1***2 **7**5*30* *1*24**423 *7*6*3**3*

            .........260.........270.........280.........290.........300
RcCYCD4_1   GIDFLEFKPS EVAAAVAISL AGETK-TVDT EKAISVLTQY VKKERMVECV
AtCYCD4_1   GIDFLEFRPS EVAAAVALSV SGELQRVHFD NSSFSPLFSL LQKERVKKIG
Consistency *******6** *******7*6 6**3504113 4464*2*243 65***52521

            .........310.........320.........330.........340.........350
RcCYCD4_1   KMVQDLSLIG ANASVPQSPI GVLDAACLSY KSDDTTVGSC PSSSNNTPDP
AtCYCD4_1   EMIESDGSDL CSQTPN--GV LEVSACCFSF KTHDSSSSYT HLS-------
Consistency 5*86404210 3535210018 0264*3*4*6 *52*552422 12*0000000

            .........360...
RcCYCD4_1   KRRKLNRPYE VEL
AtCYCD4_1   ---------- ---
Consistency 0000000000 000
```

Supplementary Fig. S4c

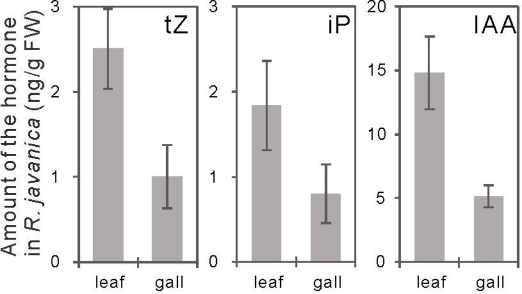

Supplementary Fig. S5
